# Treatment of refractory immune-mediated necrotizing myopathy with efgartigimod

**DOI:** 10.3389/fimmu.2024.1447182

**Published:** 2024-10-22

**Authors:** MengTing Yang, JingChu Yuan, YiKang Wang, HongJun Hao, Wei Zhang, ZhaoXia Wang, Yun Yuan, YaWen Zhao

**Affiliations:** ^1^ Department of Neurology, Peking University First Hospital, Beijing, China; ^2^ Beijing Key Laboratory of Neurovascular Disease Discovery, Beijing, China

**Keywords:** immune-mediated necrotizing myopathy, efgartigimod, refractory, anti-signal recognition particle, anti-3-hydroxy-3-methylglutaryl-CoA reductase

## Abstract

**Objective:**

We aimed to explore the efficacy and safety of efgartigimod in patients with refractory immune-mediated necrotizing myopathy (IMNM).

**Methods:**

This open-label pilot observational study included seven patients with refractory IMNM, all of whom received intravenous efgartigimod treatment. The clinical response was assessed after 4 weeks of efgartigimod treatment according to the 2016 American College of Rheumatology–European League Against Rheumatism response criteria for adult idiopathic inflammatory myopathy. Serum levels of immunoglobulin as well as anti–signal recognition particle (SRP) and anti–3-hydroxy-3-methylglutaryl-CoA reductase (HMGCR) antibodies were measured using enzyme-linked immunosorbent assays and commercial line immunoblot assays. Safety assessments included evaluations of adverse events and severe adverse events.

**Results:**

The seven patients with refractory IMNM included five cases with anti-HMGCR antibodies and two cases within anti-SRP antibodies. Four of the seven patients achieved clinical responses. The total improvement score for the responders at 4 weeks were 32.5, 40.0, 47.5, and 70.0, and those at 8 weeks were 27.5, 47.5, 57.5, and 70.0. In comparison to the responsive patients, the non-responsive patients had longer durations [8 (-) versus 2 (1–5) years, *P* = 0.03], and more chronic myopathic features by muscle biopsy (67% versus 0%, *P* = 0.046). Serum immunoglobulin G levels (11.2 ± 2.5 versus 5.7 ± 2.5, *P* = 0.007) and anti-HMGCR/SRP antibody levels (97.2 ± 6.9 versus 41.8 ± 16.8, *P* = 0.002) were decreased after treatment compared with baseline levels. Adverse events were reported in one of the seven patients, who showed mild headache.

**Conclusions:**

Despite its small size, our study demonstrated that promoting the degradation of endogenous immunoglobulin G may be effective for patients with IMNM. Efgartigimod may be a promising option for cases of refractory IMNM to shorten duration and minimize chronic myopathic features.

## 1 Introduction

Immune-mediated necrotizing myopathy (IMNM) is a major subgroup of idiopathic inflammatory myopathy characterized by severe proximal weakness and high creatine kinase (CK) levels (1–3). Based on the type of myositis-specific antibodies (MSAs) involved, IMNM can be further classified as anti–signal recognition particle (SRP) myopathy, anti–3-hydroxy-3-methylglutaryl-CoA reductase (HMGCR) myopathy, or seronegative IMNM (1, 4, 5). The relevant autoantibodies bind to target autoantigens in the muscle fibers, potentially leading to the formation of the membrane attack complex and muscle necrosis (1, 6–8).

Compared with other idiopathic inflammatory myopathy subtypes, IMNM has been considered a form of refractory myositis (9, 10), as 27% (11) to 50% (10) of patients with IMNM continue to experience severe muscle weakness even after intensive treatment. Because anti-SRP myopathy and anti-HMGCR myopathy are caused by MSAs, new biotherapies targeting B lymphocytes, such as rituximab (9, 10), ofatumumab (12), and belimumab (13), have been used to treat refractory IMNM, with positive responses in some patients. Therapeutic plasma exchange has also induced positive clinical and laboratory responses in patients with refractory IMNM (14). Those studies indicated that IMNM may benefit from rapid deletion of circulating immunoglobulin (Ig) G to remove pathogenic antibodies and improve patient symptoms.

The neonatal Fc receptor (FcRn) plays a crucial role in extending the lifespan of IgG antibodies by protecting them from lysosomal degradation and recycling them back into circulation (15, 16). Targeting this receptor could present a novel therapeutic approach for IgG-mediated diseases, as inhibiting the FcRn leads to decreased overall IgG and pathological autoantibody levels (15, 16). The development and severity of IMNM are closely linked to the presence and levels of MSAs (17, 18). A recent study showed that efgartigimod can reduce circulating IgG levels, potentially preventing further muscle necrosis and promoting muscle fiber regeneration in a mouse model of IMNM (8). These findings support the investigation of the therapeutic efficacy of efgartigimod in patients with IMNM. In this study, we evaluated the therapeutic effects of IgG reduction via efgartigimod treatment in patients with refractory IMNM.

## 2 Materials and methods

### 2.1 Patient registry

This was an observational cohort study that included seven patients who were diagnosed with IMNM according to clinical, serological, and pathological criteria (1) at the Department of Neurology at Peking University First Hospital from January to May 2024. Serum IIM antibodies, including those against Nucleosome Remodeling Deacetylase Complex Subunit Mi-2 Alpha (Mi-2α), Nucleosome Remodeling Deacetylase Complex Subunit Mi-2 Beta (Mi-2β), Transcription Intermediary Factor 1 Gamma (TIF1-γ), Melanoma Differentiation-Associated Gene 5 (MDA5), Nuclear Matrix Protein 2 (NXP2), SUMO-Activating Enzyme Subunit 1 (SAE1), Histidyl-tRNA Synthetase (Jo-1), Threonyl-tRNA Synthetase (PL-7), Alanyl-tRNA Synthetase (PL-12), Glycyl-tRNA Synthetase (EJ), Isoleucyl-tRNA Synthetase (OJ), SRP, HMGCR, Ku Autoantigen (Ku), Polymyositis-Scleroderma Autoantigen 100 kDa (PM-Scl100), Polymyositis-Scleroderma Autoantigen 75 kDa (PM-Scl75), and SSA/Ro52 Autoantigen (Ro52), were detected using Euroline Myositis Profile immunoblot assays (Euroimmun, Lubeck, Germany) according to the manufacturer’s instructions. The band intensity was reported relative to grayscale intensity as measured on a CanonScan LIDE 100 Scanner (Canon, Tokyo, Japan) using Line Scan scanning software (Euroimmun, Lubeck, Germany). The intensity of anti-SRP or anti-HMGCR antibodies in the study patients was strongly positive, with values exceeding 50. Anti-nuclear antibody was tested by an immunofluorescence assay using Hep-2010 cell line at a dilution of 1:100. Refractory criteria were defined as disease worsening or relapse after treatment with high-dose glucocorticoids and at least one immunosuppressant at a known effective dose for at least 3 months (1, 11, 19). The following exclusion criteria were applied: 1) treated with intravenous Ig or plasma exchange within the past month, and rituximab or eculizumab within the past 6 months; 2) had hepatitis virus B or C infection, other severe infection, or malignancy; 3) had low IgG serum levels (<6 g/L); 4) were pregnant, lactating, or planning to become pregnant; 5) had a history of infection requiring hospitalization within the 8 weeks prior to screening; 6) previously documented lack of clinical response to plasmapheresis; 7) vaccinated within 4 weeks before screening; or 8) had a history of malignancy. A written informed consent was obtained from all patients.

### 2.2 Data collection

Before efgartigimod treatment, we collected baseline data on the patients’ demographics, clinical manifestations, laboratory tests, electromyography results, and medication history. Serum biomarker data—including total IgG, IgA, and IgM levels, and the intensity of anti-SRP and anti-HMGCR antibodies—were also collected at baseline. Thigh muscle magnetic resonance imaging was performed on all patients before treatment. Fatty replacement of muscle was graded on T1-weighted imaging (T1WI) sequences using the scale proposed by Mercuri et al. (20), and muscle edema was graded on the basis of T2 Short Tau Inversion Recovery (T2-STIR) sequences using a four-point scale (21). Muscle biopsy was performed for all patients before treatment. Muscle specimens were assessed histologically and with immunohistochemical staining for major histocompatibility complex (MHC) class I, membrane attack complex, CD3, CD4, CD8, CD20, and CD68. To exclude various muscular dystrophies, immunohistochemical staining was performed with autoantibodies against dystrophin, α- to δ-sarcoglycans, α- and β-dystroglycans, and dysferlin.

### 2.3 Outcome assessment and response criteria

**Figure 1:**
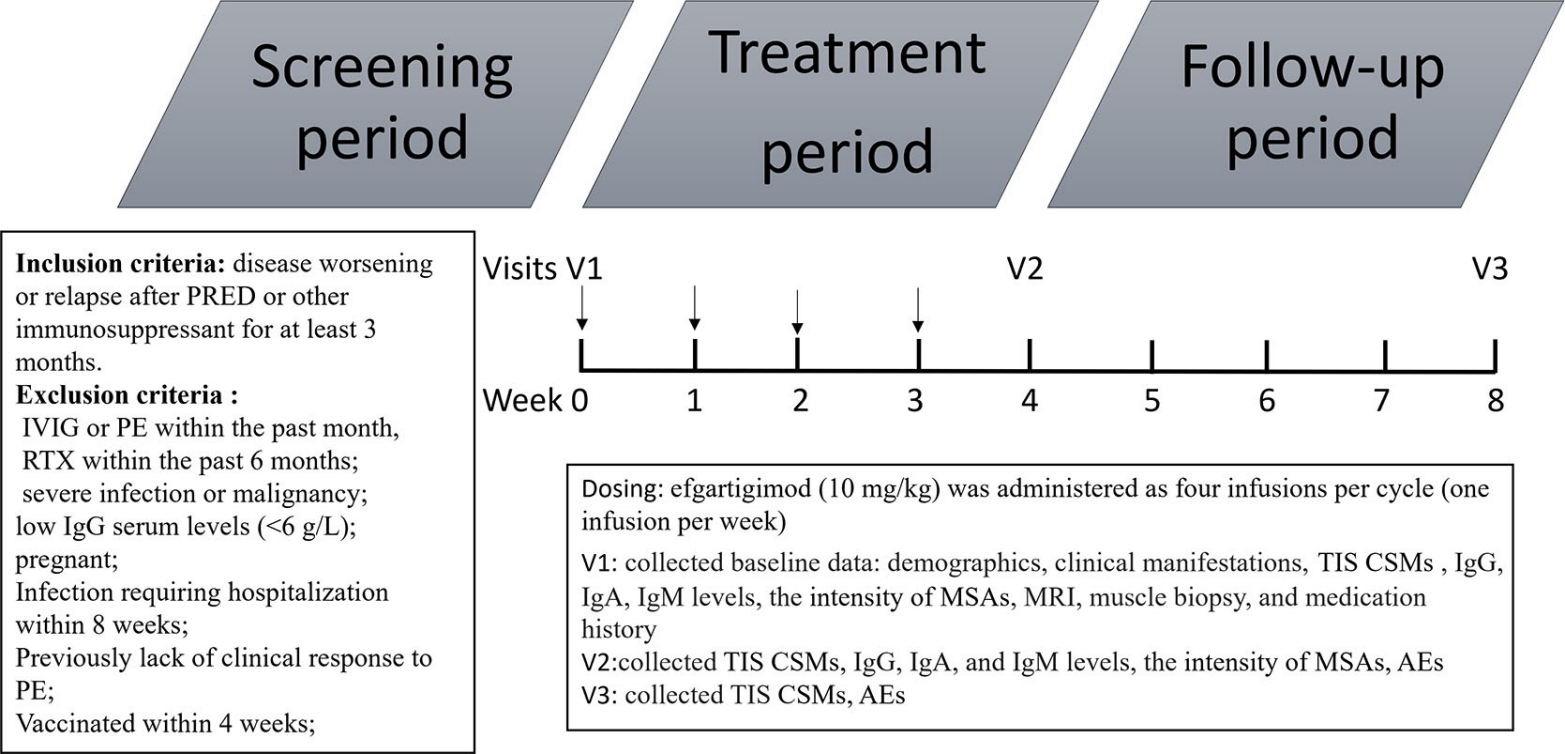
Protocol of time schedule. PRED, prednisone; IVIG, intravenous immunoglobulin; PE, plasma exchange; TIS, total improvement score; CSM, core set measures; MRI, magnetic resonance imaging; MSAs, myositis-specific antibodies AE, adverse event.

Patients were followed from the initiation of efgartigimod and through the whole treatment period of combined therapy with low-to-moderate–dose oral prednisone or tacrolimus. Three patients received low-dose prednisone (prednisone at ≤10 mg/day or equivalent) (22). Two patients received moderate-dose prednisone (prednisone at 10–30 mg/day or equivalent) (22). Four patients received tacrolimus. The concomitant oral medication regimens were unchanged during the treatment period. Efgartigimod (10 mg/kg) was administered as four infusions per cycle (one infusion per week). Clinical response was assessed using the total improvement score (TIS) according to the 2016 American College of Rheumatology–European League Against Rheumatism clinical response criteria for myositis after 4 and 8 weeks of treatment (23). The TIS (0–100) was determined by summing the scores according to the core set measures (CSMs) listed by the International Myositis Assessment and Clinical Studies Group (IMACS) to provide a quantitative measure of improvement for each patient (23). The CSMs included the Manual Muscle Testing–8 scale (MMT-8), Childhood Myositis Assessment Scale (CMAS), Physician Global Activity visual analog scale (VAS), Patient Global Activity VAS, Health Assessment Questionnaire (HAQ), Myositis Disease Activity Assessment Tool (MDAAT) Extramuscular Disease Activity VAS, and CK level. The TIS thresholds in adult patients for minimal, moderate, and major improvement were ≥20, ≥40, and ≥60 points, respectively; those in pediatric patients for minimal, moderate, and major improvement were ≥30, ≥45, and ≥70 points. The serum Ig level and MSA intensity were assessed at baseline and 4 weeks after the final infusion. Safety assessments included evaluations of adverse events (AEs), severe AEs, clinical laboratory tests, and vital signs, as well as physical examinations. The probucol of time schedule is shown in **Figure 1**.

### 2.4 Statistical analysis

Statistical analysis was performed using SPSS 26.0. Categorical variables are reported as numbers or percentages. The mean or median with standard deviation or interquartile range (IQR), respectively, was used to represent the central values of the data, depending on the normality of the distribution of the curve. We used Fisher’s exact test for comparisons of categorical variables. To compare the parameters before and after efgartigimod treatment, we use paired t-tests for comparisons of means and Wilcoxon rank sum tests for analyses of data with a non-normal distribution. Where *P* < 0.05, a difference was considered significant.

## 3 Results

### 3.1 Baseline characteristics of patients

**Figure 2:**
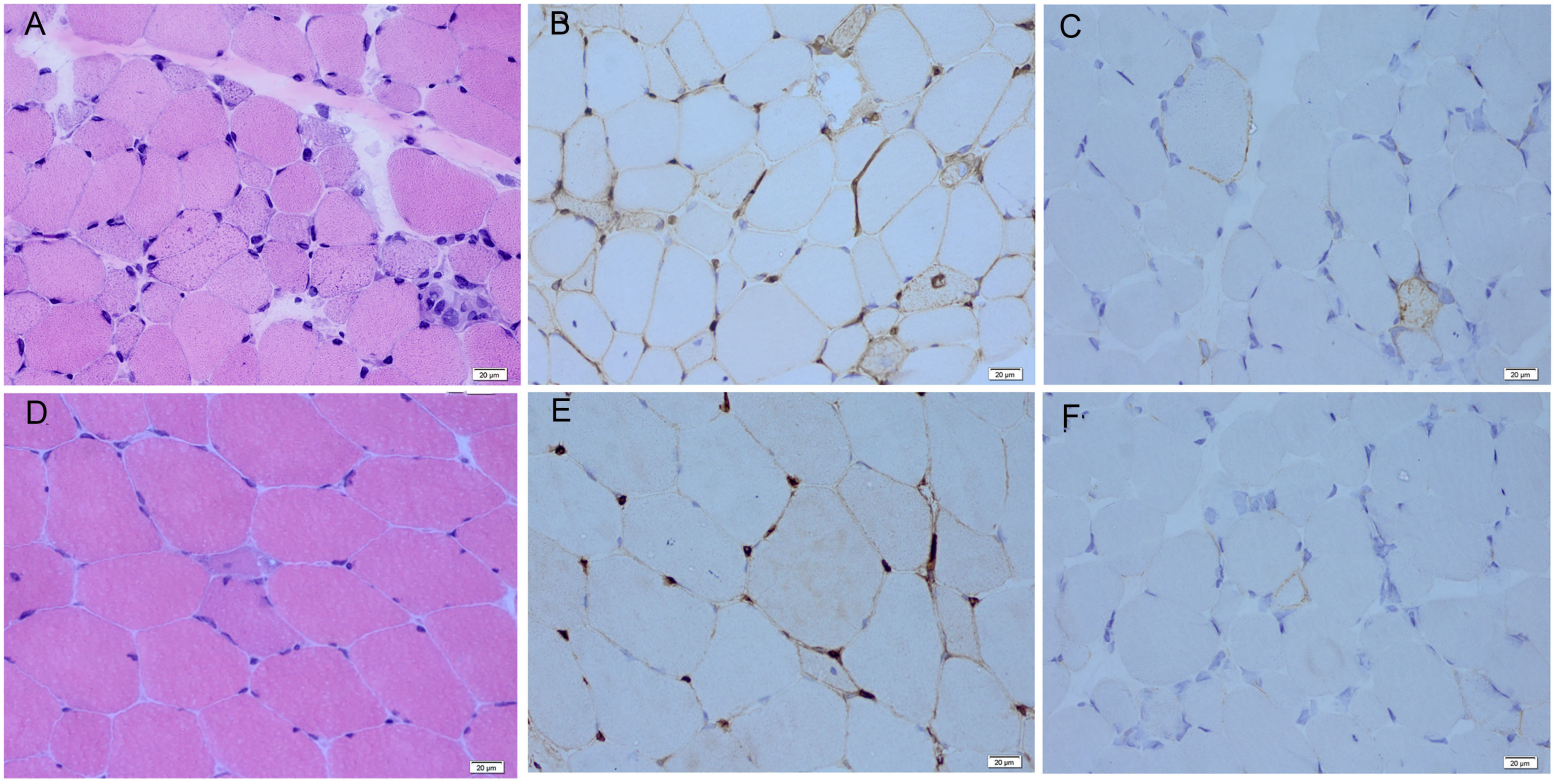
Myopathological features of responsive and non-responsive patients. **(A)** Scattered myofiber necrosis and regeneration with endomysial fibrosis are observed by hematoxylin and eosin staining in the non-responsive patients. **(B)** Diffuse sarcolemmal MHC-I deposition is seen by MHC-I immunohistochemical staining in the non-responsive patients. **(C)** Non-necrotic myofibers with little sarcolemmal MAC deposition are observed by MAC immunohistochemical staining in the non-responsive patients. **(D)** Scattered myofiber necrosis and regeneration are seen by hematoxylin and eosin staining in the responsive patients. **(E)** Diffuse sarcolemmal MHC-I deposition is observed by MHC-I immunohistochemical staining in the responsive patients. **(F)** Non-necrotic myofibers with little sarcolemmal MAC deposition are observed on MAC immunohistochemical staining in the responsive patients. MHC-I, major histocompatibility complex–I; MAC, membrane attack complex.

All patients were women, with a median age at disease onset of 21 years (10–32 years). Five patients were anti-HMGCR–positive and two patients were anti-SRP–positive (**Table 1**). The median duration of the disease was 6 years (2–8 years). All patients presented with a history of proximal muscle weakness. The median peak CK level at initial presentation was 7,234.0 IU/L (3,006.0–10,010.0). Other clinical features included myalgia in two patients, skin rashes in two patients, and muscle atrophy in two patients. Skin rashes were reported only in patients with anti-HMGCR myopathy. One patient presented with rashes on the anterior chest, which resolved spontaneously before treatment. Another patient had patchy alopecia with erythema. No patients presented with dyspnea, dysphagia, interstitial lung disease, cardiac insufficiency, Raynaud’s phenomenon, arthritis, or concomitant cancer/rheumatic disease. Anti-Ku autoantibodies were found in one patient, anti-Ro52 autoantibodies were found in one patient, and antinuclear antibodies were found in two patients. Electromyography revealed irritable myopathy changes in all patients. Muscle edema was observed in six of the seven patients by thigh muscle magnetic resonance imaging, with an average total muscle edema score of 8.1. Fatty infiltration of muscle was present in all patients, with an average total fatty infiltration score of 16.1. Muscle biopsies from all patients showed scattered necrotic and regenerating muscle fibers. Muscle biopsies from two patients, each exhibiting a dystrophic-like progression, muscle atrophy, and severe fatty replacement in MRI, also revealed chronic myopathic features with endomysial fibrosis and greater variations in fiber size (**Figure 2**). All patients were initially treated with high-dose prednisone and received various additional immunotherapies for 5 years (2–8 years), including methotrexate in five, tacrolimus in five, azathioprine in two, cyclophosphamide in two, intravenous Ig in five, rituximab in four, and ofatumumab in two.

**Table 1:** Baseline demographics and clinical characteristics of patients with IMNM.

	Patient 1	Patient 2	Patient 3	Patient 4	Patient 5	Patient 6	Patient 7
Sex	Female	Female	Female	Female	Female	Female	Female
MSAs	HMGCR	HMGCR	SRP	HMGCR	HMGCR	SRP	HMGCR
Age at onset, years	13	21	32	5	25	56	10
Duration, years	7	6	8	23	2	1	2
Muscle weakness	+	+	+	+	+	+	+
Myalgia	−	−	−	−	+	+	−
Muscle atrophy	−	+	−	+	−	−	−
Skin rashes	+	−	−	+	−	−	−
Peak CK level, IU/L	2034	7,000	10,121	3,006	13,537	7,234	10,010
EMG	+	+	+	+	+	+	+
Muscle edema in MRI	0	16	5	8	11	9	8
Fatty infiltration in MRI	3	31	34	35	3	4	3
Muscle biopsy	Necrosis pattern	Chronic pattern	Necrosis pattern	Chronic pattern	Necrosis Pattern	Necrosis Pattern	Necrosis Pattern
Previous medication	PRED, MTX, TAC, and IVIG	PRED, MTX, TAC, CTX, IVIG, RTX, and OFA	MTX, Aza, CTX, and IVIG	PRED, MTX, Aza, TAC, RTX, and OFA	PRED, TAC, IVIG, and RTX	PRED and MTX	PRED, TAC, IVIG, and RTX

IMNM, immune-mediated necrotizing myopathy; HMGCR, 3-hydroxy-3-methylglutaryl-CoA reductase; I SRP, signal recognition particle; CK, creatine kinase; EMG, electromyogram; MRI, magnetic resonance imaging; PRED, prednisone; MTX, methotrexate; CTX, cyclophosphamide; TAC, tacrolimus; Aza, azathioprine; OFB, ofatumumab; RTX, rituximab; IVIG, intravenous immunoglobulin.

### 3.2 Clinical response to treatment

**Figure 3:**
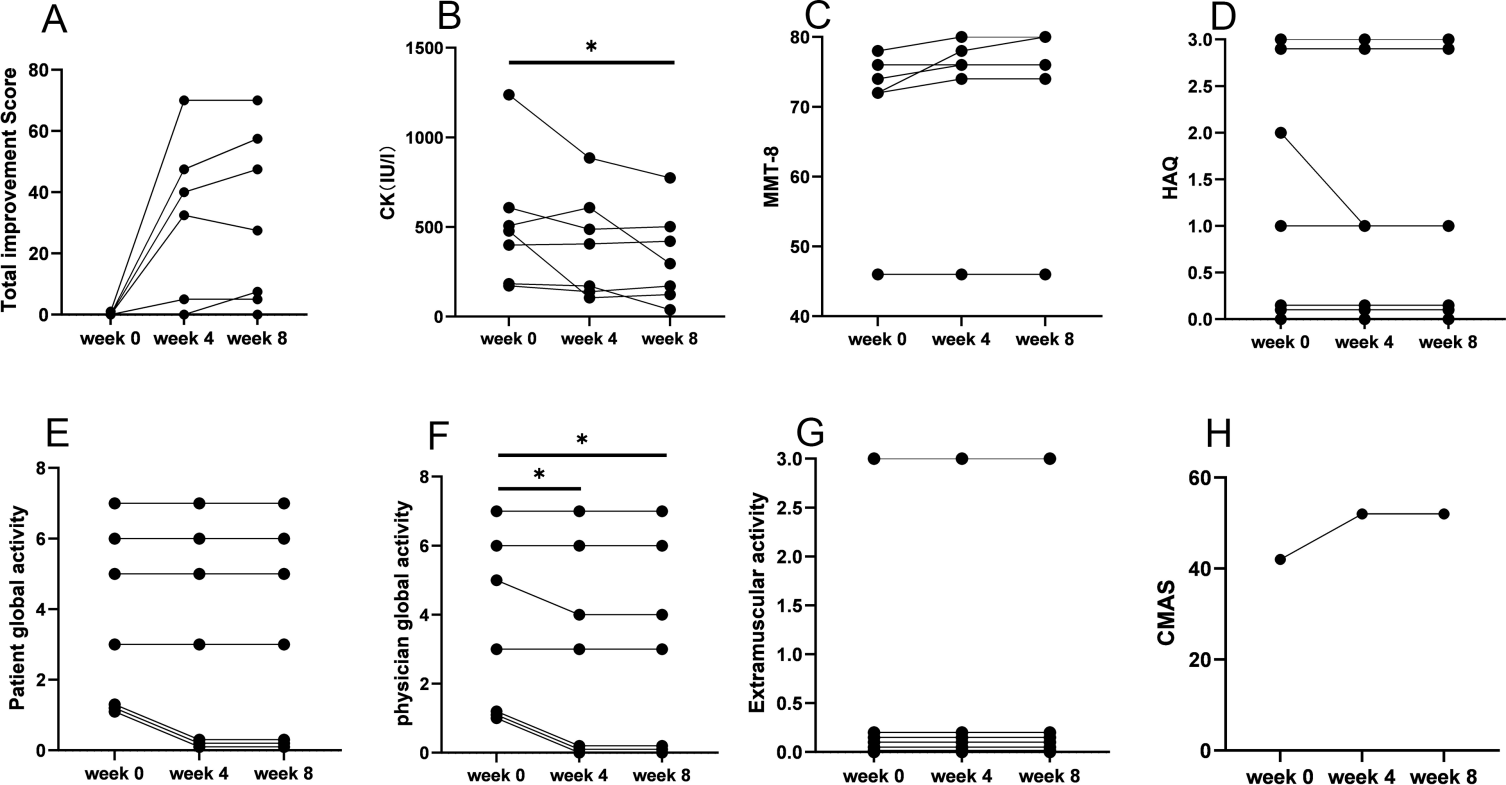
IMACS CSM in patients with IMNM at baseline and after treatment with efgartigimod. **(A)** Total improvement score. **(B)** CK level. **(C)** MMT-8 score. **(D)** HAQ score. **(E)** Patient Global Activity. **(F)** Physician Global Activity. **(G)** Extramuscular activity. **(H)** CMAS score. IMACS, International Myositis Assessment and Clinical Studies Group; CSMs, core set measures; IMNM, immune-mediated necrotizing myopathy; CK, creatine kinase; MMT-8, Manual Muscle Testing–8; HAQ, Health Assessment Questionnaire; CMAS, Childhood Myositis Assessment Scale; *P < 0.05.

Efgartigimod demonstrated early disease control in four of the seven (57%) patients within 4 weeks of treatment. Four patients (one with anti-HMGCR and three with anti-SRP antibodies) attained minimal to major improvement in 4 weeks, which persisted 8 weeks after efgartigimod treatment. The TIS for the responders at 4 weeks were 32.5, 40.0, 47.5, and 70.0, and those at 8 weeks were 27.5, 47.5, 57.5, and 70.0 (**Figure 3**). Physician Global Activity [3.0 (IQR, 1.0–5.0) versus 3.0 (IQR, 0.0–5.0), P = 0.046] at 4 weeks after treatment was significantly better than that in baseline. There were statistically significant improvements at 8 weeks after treatment compared with baseline in the following CSMs (**Figure 1**, **Supplementary Table S1**): Physician Global Activity [3.0 (IQR, 1.0–5.0) versus 3.0 (IQR, 0.0–5.0), *P* = 0.046] and CK levels [478.0 (184.0–608.0) versus 296.0 (123.0–502.0) IU/L, *P* = 0.04]. Other CSMs—such as MMT-8, CMAS, Patient Global Activity VAS, HAQ, and Extramuscular Disease Activity—showed no significant improvement 4 or 8 weeks after treatment (**Figure 3**, **Supplementary Table S1**). In comparison to the responsive patients, the non-responsive patients had longer durations [8 (-) versus 2 (1–5) years, *P* = 0.03] and more chronic myopathic features by muscle biopsy (67% versus 0.0%, *P* = 0.046) (**Figure 2**, **Supplementary Table S2**). Subgroup analysis indicated that the beneficial effects of efgartigimod were evident regardless of autoantibody status and dosage of steroids and/or additional non-steroidal immunosuppressive drugs (**Supplementary Table S3**).

### 3.3 Serum Igs and anti-desmoglein antibody levels

**Figure 4:**
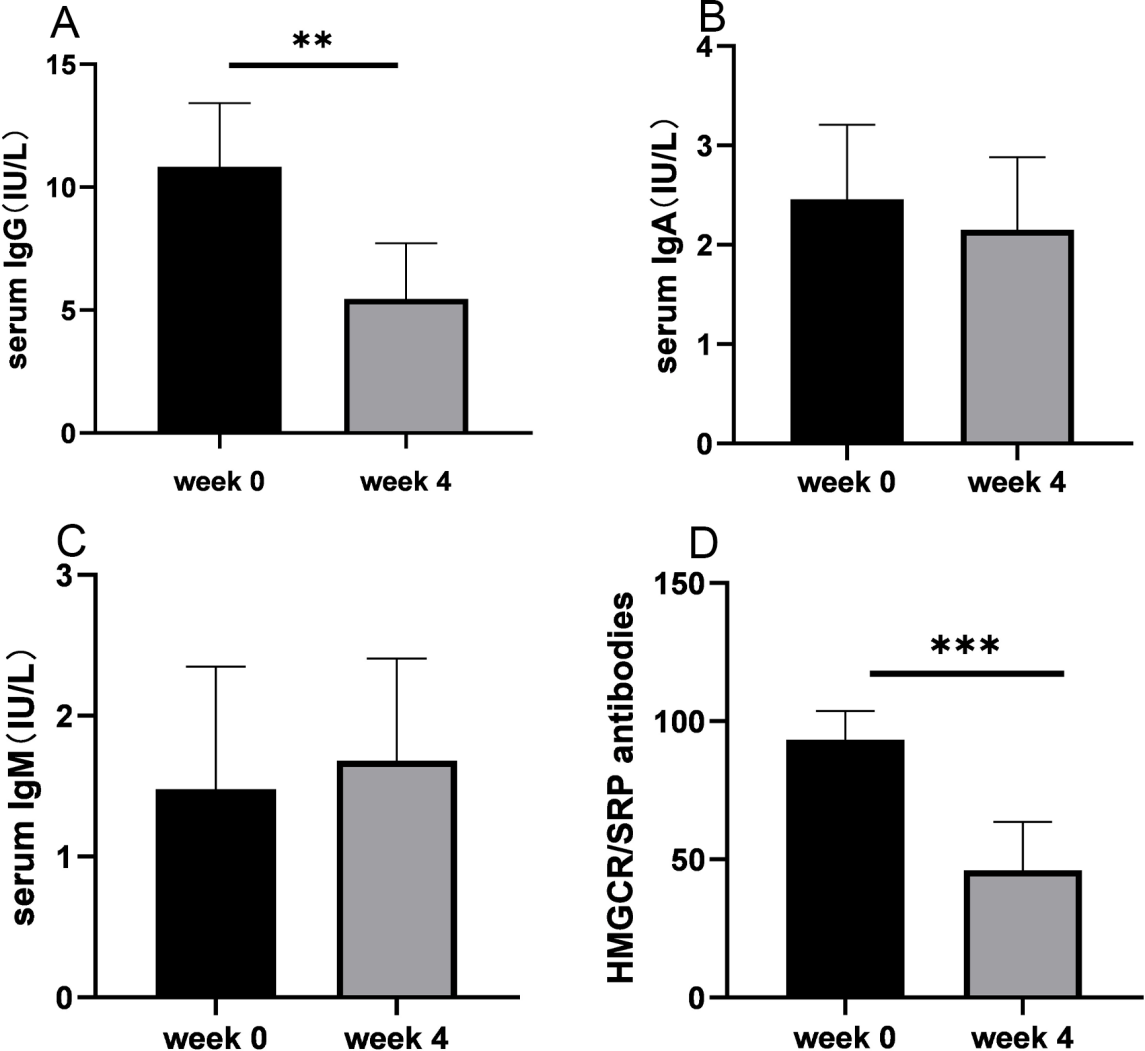
Serum levels of immunoglobulins and autoantibodies before and after treatment with efgartigimod. **(A)** Serum IgG levels were significantly decreased after treatment compared with baseline levels. **(B)** Serum IgA levels showed no significant difference after treatment. **(C)** Serum IgM levels showed no significant difference after treatment. **(D)** Staining intensity of anti-HMGCR/SRP antibodies was significantly reduced after treatment compared with baseline staining. Ig, immunoglobulin; HMGCR, 3-hydroxy-3-methylglutaryl-CoA reductase; SRP, signal recognition particle. **P < 0.01 and ***P < 0.005.

Serum IgG levels significantly decreased after treatment compared with baseline levels (11.2 ± 2.5 versus 5.7 ± 2.5, *P* = 0.007; **Figure 4A**), with no differences observed between responders and non-responders. Serum IgG levels decreased from baseline for anti-HMGCR myopathy (mean, 38%) and anti-SRP myopathy (mean, 53%) at the end of the induction phase. There were no clinically relevant changes from the baseline levels of IgA and IgM (**Figures 4B, C**). MSA intensity significantly decreased post-treatment compared with baseline (97.2 ± 6.9 versus 41.8 ± 16.8, *P* = 0.002; **Figure 4D**), with no distinction between responders and non-responders. The intensity of HMGCR antibodies decreased by a mean of 56% from baseline and 60% for SRP antibodies at the end of the induction phase. Subgroup analysis indicated that the changes in serum IgG levels and antibody levels were evident regardless of concomitant medications (**Supplementary Table S3**).

### 3.4 Safety data

During the 8-week study period, AEs were reported in one of the seven patients on efgartigimod, who experienced mild headache. A slightly abnormal differential leukocyte count was detected in one case. None of the patients required efgartigimod dose reduction or withdrawal owing to AEs. There were no severe AEs (**Table 2**).

**Table 2:** Summary of AEs in all patients.

AEs	Number (n = 7)
Any AEs	1/7
Any severe AEs	0/7
Any AEs leading to discontinuation of study drug	0/7
Infusion-related reaction event	0/7
Most common adverse events	1/7
Headache	1/7
Nasopharyngitis	0/7
Nausea	0/7
Diarrhea	0/7
Upper respiratory tract infection	0/7
Urinary tract infection	0/7

AEs, adverse events.

## 4 Discussion

We present a single-center, retrospective case series using efgartigimod for refractory IMNM. The seven patients with refractory IMNM included five cases with anti-HMGCR antibodies and two cases within anti-SRP antibodies. All patients presented with proximal muscle weakness and high CK levels. Extramuscular symptoms included skin rashes in two cases with anti-HMGCR antibodies. The Dermatomyositis (DM)-like rashes have been reported in anti-HMGCR myopathy with a frequency ranging from 38% (24) to 43% (25) in previous studies. Despite multiple immunosuppressants, all patients had precipitous declines in strength and quality of life, which led to a trial of efgartigimod treatment.

Although the beneficial effect of FcRn antagonism in refractory IMNM may be attributable to a combination of mechanisms, strategies to deplete pathogenic antibodies have been shown to have a profound impact on patients’ responses to therapy. In our study, four patients experienced rapid symptomatic improvement within 4 weeks of efgartigimod treatment. The initial clinical improvement after efgartigimod was dramatic, similar to results in the context of myasthenia gravis (16). Moreover, these improvements persisted even 4 weeks after discontinuation of efgartigimod, indicating that its therapeutic effects are durable. Physician global activity and serum CK levels were significantly improved after treatment, whereas extramuscular symptom (skin rashes) showed no improvement. The concomitant oral medication regimens, which included prednisone and tacrolimus, were low and unchanged during treatment, as was reported in previous studies (26, 27). We evaluated whether the benefits of efgartigimod were consistent across key patient clinical characteristics. Disease control was similar in patients regardless of autoantibody status and/or concomitant medications, suggesting efgartigimod contributed to clinical efficacy.

Patients with a poor outcome in our study had longer durations and more chronic myopathic features by muscle biopsy. In the setting of chronic muscle damage, immune dysregulation and abnormal fibro-adipogenic progenitor differentiation can occur, leading to differentiation into fat cells or fibroblasts, progressive tissue fibrosis, and loss of normal tissue architecture, ultimately causing irreversible damage to the muscle (28). Therefore, although there is no consensus protocol for efgartigimod in IMNM, we suggest that an initial trial of efgartigimod for early-stage disease should be considered. Clinicopathological changes should be considered during patient selection. Muscular dystrophy–like pathology should be an exclusion criterion in further studies, as those pathological changes are currently untreatable.

The pharmacokinetic parameters in this study (10 mg/kg) were in line with data from other studies (15, 27). Efgartigimod rapidly decreased circulating IgG levels from baseline in patients, including autoantibodies, which has also been reported in myasthenia gravis and primary immune thrombocytopenia (16, 29). During the efgartigimod induction phase, early reductions of approximately 50% from baseline in total serum IgG and anti-SRP/HMGCR antibodies were observed after 4 weeks of treatment. Julien et al. also reported that administration of efgartigimod could decrease IgG levels and anti-HMGCR antibodies to prevent further necrosis and allow muscle fiber regeneration in a humanized mouse model of IMNM (8). It is noteworthy that both total IgG and pathogenic antibodies levels were reduced in non-responsive patients, suggesting that these patients may have disease with a non-IgG–mediated mechanism. We found the serum levels of IgA and IgM are not affected by efgartigimod, which has also been reported in myasthenia gravis and healthy volunteers (30, 31). These data reflect the mechanism of efgartigimod action of selective IgG reduction, which leads to incomplete IgG reduction without altering other Ig levels (31, 32).

The primary outcome of the study was safety, and efgartigimod was well tolerated, with few AEs. Mild headache is a well-known side effect of efgartigimod treatment and was reported in 16% of patients with primary immune thrombocytopenia (29) and in 29% of patients with myasthenia gravis (16). Most AEs resolve spontaneously or rapidly upon treatment without the need to discontinue efgartigimod (29). Transient decreases in blood leukocyte levels were observed and were also found in 7 of the 20 healthy volunteers (31). Several studies presented upper respiratory tract infections and urinary tract infections (30); however, a higher rate of infection was not observed in our patients. The efgartigimod did not inhibit production of protective IgG and the risk of infections is unaltered during efgartigimod treatment (31).

Our study preliminarily explored the efficacy and safety of efgartigimod in patients with refractory IMNM. However, all participants underwent only a single-treatment cycle, which raises uncertainty regarding the sustainability of efgartigimod therapy in this patient population. To enhance therapeutic outcomes, it may be advantageous to adopt a sequential treatment approach with efgartigimod aimed at achieving sustained reductions in IgG levels. The ADVANCE study showed the effectiveness and well toleration of efgartigimod using a treatment regimen of either once per week or biweekly for adults with primary immune thrombocytopenia (29). The median interval between treatment cycles in the ADAPT (16) and ADAPT+ (33) studies, which was determined by clinical evaluation of each participant with myasthenia gravis, was approximately 5.8 to 7.3 weeks. Thus, regular monitoring of IgG levels, clinical symptoms, and AEs is essential to identify the optimal timing for subsequent doses during efgartigimod treatment for IMNM. Additionally, previous research has indicated that combination therapy with telitacicept and the faster-acting efgartigimod may represent an effective and safe therapeutic approach for refractory myasthenia gravis (34). Given the close association of IMNM with antibody-mediated pathogenesis, B-cell–targeting treatments to suppress antibody production could also be complementary to efgartigimod (1).

There are several limitations to this study. First, the majority of study participants had previously received various third-line treatments with poor outcomes; therefore, the results may not be generalizable to treatment-naïve patients with IMNM. Second, another mitigating factor is the time from diagnosis to initial treatment with efgartigimod, as well as the duration of acute decline in strength, both of which may mark more extensive muscle damage that may not be reversible by reducing pathologic antibody levels. Some participants included may have been too far advanced in the course of the disease to respond to efgartigimod. Third, small sample size and short observation period limit the ability to evaluate sustained efficacy and rare AEs and may not adequately represent the broader patient population. Finally, we used commercial line immunoblot assay to observe the relative levels of HMGCR and SRP antibodies due to technical factor. We suggest the importance of establishing available titer assays such as quantitative Enzyme linked immunosorbent assay (ELISA) for HMGCR and SRP antibodies, which may be better to track the efficacy of efgartigimod. Future studies are necessary to evaluate the effectiveness of efgartigimod for IMNM more systematically, which may entail establishing a registry of IMNM patient cases and large, prospective studies to assess clinical outcomes using a standardized approach with defined biomarkers and validated clinical endpoints.

In conclusion, to our knowledge, this is the first study evaluating the efficacy and safety of an FcRn inhibitor for the treatment of refractory IMNM. Our findings suggest that efgartigimod may be an encouraging option for refractory IMNM cases. Although a prospective clinical trial remains to be performed, our study demonstrated that promoting the degradation of endogenous IgG may be effective for patients with IMNM, which may pave the way for the efficient design of future trials in idiopathic inflammatory myopathy.

## Data Availability

The original contributions presented in the study are included in the article/**Supplementary Material**. Further inquiries can be directed to the corresponding author.
