# Supplementary material for: Treatment of refractory immune-mediated necrotizing myopathy with efgartigimod
Source: Front Immunol. 2024 Oct 22;15:1447182. doi: 10.3389/fimmu.2024.1447182 (PMC11534618; doi:10.3389/fimmu.2024.1447182)
Supplement: Supplementary file 1 [file Table1.docx]

Table S1. IMACS CSMs in patients at the baseline and after treatment.

| Characteristics | Week 0 | Week 4 | Week 8 | P value(Week 0 *VS* Week 4) | P value (Week 0 *VS* Week 8) |
| --- | --- | --- | --- | --- | --- |
| Physician global activity, median（IQR） | 3.0(1.0-5.0) | 3.0(0.0-5.0) | 3.0(0.0-5.0) | 0.046* | 0.046* |
| Patient global activity, median（IQR） | 3.0(1.0-6.0) | 3.0(0.0-6.0) | 3.0(0.0-6.0) | 0.08 | 0.08 |
| MMT-8, mean±SD | 70.0±10.8 | 72.0±11.8 | 72.6±12.1 | 0.11 | 0.08 |
| HAQ, median（IQR） | 0.0(0.0-3.0) | 0.0(0.0-3.0) | 0.0(0.0-3.0) | 1.00 | 1.00 |
| CK, median（IQR）, IU/L | 478.0(184.0-608.0) | 406.0(139.0-608.0) | 296.0(123.0-502.0) | 0.12 | 0.04* |
| Extramuscular activity, median（IQR） | 0.0(0.0-0.0) | 0.0(0.0-0.0) | 0.0(0.0-0.0) | 1.00 | 1.00 |
| CMAS score | 42(-) | 52(-) | 52(-) | - | - |
| Serum IgA level，mean±SD，IU/L | 2.5±0.7 | 2.2±0.7 | - | 0.57 | - |
| Serum IgM level，mean±SD，IU/L | 1.5±0.7 | 1.7±0.7 | - | 0.61 | - |
| Serum IgG level，mean±SD，IU/L | 11.2±2.5 | 5.7±2.5 | - | 0.007* | - |
| Intensity of HMGCR/SRP antibodies，mean±SD | 97.2±6.9 | 41.8±16.8 | - | 0.002* | - |

MACS: International Myositis Assessment and Clinical Studies Group; CSM: core set measures; IIMNM: immune-mediated necrotizing myopathy; SD：standard deviation; IQR: interquartile range; MMT-8: manual muscle testing -8; HAQ: Health Assessment Questionnaire; CK: creatine kinase; CMAS: Childhood Myositis Assessment Scale; Ig: immunoglobulin; HMGCR: 3-hydroxy-3-methylglutaryl-coA reductase; I SRP: signal recognition particle; *p < 0.05;
