# Supplementary material for: Treatment of refractory immune-mediated necrotizing myopathy with efgartigimod
Source: Front Immunol. 2024 Oct 22;15:1447182. doi: 10.3389/fimmu.2024.1447182 (PMC11534618; doi:10.3389/fimmu.2024.1447182)
Supplement: Supplementary file 2 [file Table2.docx]

Table S2. Clinical features of the responsive and no responsive patients.

| Characteristics | Responsive patients (n=4) | No responsive patients(n=3) | P value |
| --- | --- | --- | --- |
| Female, n(%) | 4(100%) | 3(100%) | 1.00 |
| Anti-HMGCR positive, n(%) | 3(60%) | 1(67%) | 1.00 |
| Age at symptom onset, median,(IQR), years | 23(13-48) | 13(-) | 1.00 |
| Duration, median (IQR), years | 2(1-5) | 8(-) | 0.03* |
| Skin rashes, n(%) | 1(25%) | 1(33%) | 1.00 |
| Muscle odema score, median (IQR) | 10.0(8.3-14.8) | 5.0(-) | 0.16 |
| Fatty infiltration score, median (IQR) | 10.0(3.5-24.3) | 24.0(-) | 0.14 |
| Chronic myopathy pattern in biopsy ,n(%) | 0(0.0%) | 2(67.0%) | 0.046* |
| Dose of additional steroids, median (IQR) | 15.0(2.5-27.5) | 7.5(-) | 0.43 |
| Additional immunotherapies，n(%) | 2(50%) | 2(67%) | 1.00 |
| Physician global activity at baseline, median（IQR） | 1.0(1.0-4.0) | 5.0(-) | 0.49 |
| Patient global activity at baseline, median（IQR） | 1.0(1.0-4.0) | 6.0(-) | 0.49 |
| MMT-8 at baseline, median（IQR） | 73.0(72.0-77.0) | 72.0(-) | 1.00 |
| HAQ at baseline, median（IQR） | 0.5(0.0-2.5) | 2.0(-) | 1.00 |
| CK at baseline, median（IQR）, IU/L | 493.0(248.5-1055.5) | 400.0(-) | 1.00 |
| Extramuscular activity at baseline, median（IQR） | 0.0(0.0-0.0) | 0.0(-) | 1.00 |
| Serum IgG level at baseline，median（IQR），IU/L | 9.5(8.4-13.4) | 12.7(-) | 1.00 |
| Intensity of anti-HMGCR/SRP antibodies at baseline，median（IQR） | 100(87.3-100) | 100(-) | 1.00 |

IMNM: immune-mediated necrotizing myopathy; HMGCR: 3-hydroxy-3-methylglutaryl-coA reductase; I SRP: signal recognition particle; SD：standard deviation; IQR: interquartile range; IMNM: immune-mediated necrotizing myopathy; CK: creatine kinase; MMT-8: manual muscle testing -8; HAQ: Health Assessment Questionnaire; CMAS: Childhood Myositis Assessment Scale; *p < 0.05;
