# Supplementary material for: Treatment of refractory immune-mediated necrotizing myopathy with efgartigimod
Source: Front Immunol. 2024 Oct 22;15:1447182. doi: 10.3389/fimmu.2024.1447182 (PMC11534618; doi:10.3389/fimmu.2024.1447182)
Supplement: Supplementary file 3 [file Table3.docx]

Table S3. Subgroup analysis

|  | Autoantibodies | | Dosage of prednisone | | | Additional immunotherapies (tacrolimus) | |
| --- | --- | --- | --- | --- | --- | --- | --- |
|  | Anti-SRP+(n=2) | Anti-HMGCR+(n=5) | None (0 mg, n=2) | Low(＜10mg，n=3) | Moderate(10-30mg，n=2) | Yes (n=4) | No (n=3) |
| Responder at Week 4 and Week 8, n (%) | | | | | | | |
| Week 4 | 1(50%) | 3(60%) | 2(100%) | 1(33%) | 1(50%) | 2(50%) | 2(67%) |
| Week 8 | 1(50%) | 3(60%) | 2(100%) | 1(33%) | 1(50%) | 2(50%) | 2(67%) |
| Percent change from baseline in physician global activity at Week 4 and Week 8, Mean ±SD | | | | | | | |
| Week 4 | 50.0±70.7 | 40.0±54.8 | 50.0±70.7 | 33.3±57.7 | 60.0±56.6 | 50.0±57.7 | 40.0±52.9 |
| Week 8 | 50.0±70.7 | 44.0±51.8 | 50.0±70.7 | 33.3±57.7 | 60.0±56.6 | 50.0±57.7 | 40.0±52.9 |
| Percent change from baseline in patient global activity at Week 4 and Week 8, Mean ±SD | | | | | | | |
| Week 4 | 50.0±70.7 | 40.0±54.8 | 50.0±70.7 | 33.3±57.7 | 50.0±70.7 | 50.0±57.7 | 33.3±57.7 |
| Week 8 | 50.0±70.7 | 40.0±54.8 | 60.0±56.6 | 33.3±57.7 | 50.0±70.7 | 50.0±57.7 | 33.3±57.7 |
| Percent change from baseline in MMT-8 at Week 4 and Week 8, Mean ±SD | | | | | | | |
| Week 4 | 5.6±7.8 | 6.6±11.9 | 15.2±17.7 | 3.7±6.4 | 1.4±1.9 | 1.3±1.5 | 12.9±13.9 |
| Week 8 | 5.6±7.8 | 7.7±11.7 | 15.2±17.7 | 3.7±6.4 | 4.1±5.7 | 2.7±3.9 | 12.9±13.9 |
| Percent change from baseline in HAQ at Week 4 and Week 8, Mean ±SD | | | | | | | |
| Week 4 | 0.0±0.0 | 0.0±0.0 | 0.0±0.0 | 0.0±0.0 | 0.0±0.0 | 0.0±0.0 | 0.0±0.0 |
| Week 8 | 0.0±0.0 | 0.0±0.0 | 0.0±0.0 | 0.0±0.0 | 0.0±0.0 | 0.0±0.0 | 0.0±0.0 |
| Percent change from baseline in CK at Week 4 and Week 8, Mean ±SD | | | | | | | |
| Week 4 | 7.5±0.5 | 9.3±19.6 | 23.9±6.6 | 4.5±5.2 | 0.1±28.0 | 8.5±10.4 | 9.0±21.0 |
| Week 8 | 76.9±3.0 | 18.3±21.1 | 19.5±26.1 | 49.5±47.5 | 30.0±17.2 | 23.3±44.6 | 43.9±25.7 |
| Percent change from baseline in extramuscular activity at Week 4 and Week 8, Mean ±SD | | | | | | | |
| Week 4 | 0.0±0.0 | 0.0±0.0 | 0.0±0.0 | 0.0±0.0 | 0.0±0.0 | 0.0±0.0 | 0.0±0.0 |
| Week 8 | 0.0±0.0 | 0.0±0.0 | 0.0±0.0 | 0.0±0.0 | 0.0±0.0 | 0.0±0.0 | 0.0±0.0 |
| Percent change from baseline in serum IgG, Mean ±SD | 37.5±14.6 | 53.0±14.3 | 58.4±12.4 | 63.9±14.6 | 37.2±0.5 | 61.2±3.9 | 37.2±0.5 |
| Percent change from baseline in autoantibody，Mean ±SD | 60.0±5.7 | 56.3±23.1 | 48.0±25.5 | 56.0±12.3 | 57.8±16.7 | 50.7±18.6 | 68.5±6.4 |

HMGCR: 3-hydroxy-3-methylglutaryl-coA reductase; SRP: signal recognition particle; SD：standard deviation; MMT-8: manual muscle testing -8; HAQ: Health Assessment Questionnaire;

CK: creatine kinase; Ig：immunoglobulin
